# Diminution of Heart Rate Variability in Bipolar Depression

**DOI:** 10.3389/fpubh.2017.00312

**Published:** 2017-12-06

**Authors:** Brandon Hage, Briana Britton, David Daniels, Keri Heilman, Stephen W. Porges, Angelos Halaris

**Affiliations:** ^1^Department of Psychiatry and Behavioral Neurosciences, Stritch School of Medicine, Loyola University Chicago, Maywood, IL, United States; ^2^Department of Psychiatry, University of North Carolina, Chapel Hill, NC, United States; ^3^Kinsey Institute, Indiana University Bloomington, Bloomington, IN, United States

**Keywords:** major depression, heart rate variability, respiratory sinus arrhythmia, escitalopram, celecoxib

## Abstract

Autonomic nervous system (ANS) dysregulation in depression is associated with symptoms associated with the ANS. The beat-to-beat pattern of heart rate defined as heart rate variability (HRV) provides a noninvasive portal to ANS function and has been proposed to represent a means of quantifying resting vagal tone. We quantified HRV in bipolar depressed (BDD) patients as a measure of ANS dysregulation seeking to establish HRV as a potential diagnostic and prognostic biomarker for treatment outcome. Forty-seven BDD patients were enrolled. They were randomized to receive either escitalopram–celecoxib or escitalopram-placebo over 8 weeks in a double-blind study design. Thirty-five patients completed the HRV studies. Thirty-six healthy subjects served as controls. HRV was assessed at pretreatment and end of study and compared with that of controls. HRV was quantified and corrected for artifacts using an algorithm that incorporates time and frequency domains to address non-stationarity of the beat-to-beat heart rate pattern. Baseline high frequency-HRV (i.e., respiratory sinus arrhythmia) was lower in BDD patients than controls, although the difference did not reach significance. Baseline low-frequency HRV was significantly lower in BDD patients (ln4.20) than controls (ln = 5.50) (*p* < 0.01). Baseline heart period was significantly shorter (i.e., faster heart rate) in BDD patients than controls. No significant change in HRV parameters were detected over the course of the study with either treatment. These findings suggest that components of HRV may be diminished in BDD patients.

## Introduction

Compared to major depressive disorder (MDD) and anxiety disorders, bipolar disorder (BD) is less prevalent, but it represents a significant mental health concern worldwide. BD is one of the most burdensome mental illnesses worldwide with nearly 50 million people suffering from it (1). Although manic episodes represent the distinguishing feature for a diagnosis of BD type I versus other mood disorders, bipolar depression (BDD) causes significant distress and dysfunction for patients and their families and poses major treatment challenges. Because it is often difficult to distinguish BDD from an episode of MDD prior to a distinct episode of mania, it poses significant diagnostic challenges to the clinician. Although episode length and frequency of episodes may be relatively similar between depression and mania in BD, clinical evidence suggests that BD patients are less likely to fully recover from a major depressive episode than from a manic, hypomanic, or even minor depressive episode (2) Therefore, it is imperative to consider BDD patients as being particularly susceptible to treatment resistance. Many symptoms of mood disorders may be reflective of an underlying dysregulation in autonomic nervous system (ANS) function. For example, the increased sympathetic activity with associated elevations in catecholamine and cortisol levels observed in anxiety results in a high comorbidity between anxiety and depression (3). This sustained increase in sympathetic tone can result in changes in blood pressure, decreased blood flow to the gastrointestinal tract leading to weight loss, and insomnia due to sustained pupillary dilation.

Heart rate variability (HRV) is defined as the variation between heartbeats over a period of time; it involves input from both the sympathetic and parasympathetic divisions of the ANS. Short recordings on an electrocardiogram (ECG) produce two primary patterns of oscillation that correspond to HRV (4). One frequency band occurs between about 2 and 8 s (approximately one breath cycle in the general population), which corresponds to 0.12–0.4 Hz (high frequency, or HF-HRV). This oscillation coincides with a physiological phenomenon known as respiratory sinus arrhythmia (RSA), which is characterized by a spontaneous oscillation in the beat-to-beat heart rate pattern that occurs in relation to spontaneous breathing. It is accepted throughout the literature that RSA, or HF-HRV, can be used to estimate cardiac vagal tone (5, 6). A second frequency band occurs between about 10 and 25 s, which corresponds to 0.04–0.10 Hz (low frequency, or LF-HRV). The pattern produced by this frequency band is often known as the Traube–Hering–Mayer wave. Certain studies have attempted to validate the LF measurement as an index of sympathetic activity (7, 8), while other evidence suggests that LF measurements are more reflective of mixed sympathetic and parasympathetic activity (9, 10). A third theory suggests that since atropine, a cholinergic blocker, removes both oscillations in heart rate, the LF-HRV domain is also transmitted through the vagus nerve and represents *another* parasympathetic index (11). Given that abundant evidence has validated RSA as an index of cardiac vagal tone (12) and since there is substantial ambiguity in interpreting other components of HRV (13, 14), we chose to use RSA (i.e., HF-HRV) as our primary index of autonomic function and LF-HRV as a more general index of autonomic state *via* a pathway that is presently not well defined.

The comorbidity between affective disorders and cardiovascular (CVD) and cerebrovascular disease is well documented in the literature. Elucidating the likely pathophysiological links between CVD and mental illness has been a major research focus over the past several decades, and there is growing evidence indicating that one of these links may be HRV. Studies indicate that decreased HRV may be indicative of a myocardial infarction (15, 16) and insulin resistance (17). The relationship between MDD and CVD has been robustly established in the literature (18–20). A decreased RSA and an increased LF-HRV has been associated with MDD (21), and further evidence suggests that increased RSA prior to antidepressant treatment is predictive of a positive treatment response in MDD patients.^1^ The literature associating HRV to BD is not as robust, but recent research suggests links between the two (22). In one particular study, both BD and recurrent MDD patients were found to have significantly lower HRV parameters than healthy controls (HCs), despite clinical remission in both groups (23). In BD patients, HRV during a manic episode is significantly higher than HRV during a depressive episode or a euthymic state (24, 25). In the present study, we addressed three specific aims. First, we sought to detect differences in HRV between BDD patients and HC subjects before the initiation of antidepressant drug therapy in BDD patients with documented treatment resistance in regards to depression response. Second, based on our previous findings of treatment response prediction utilizing baseline RSA values in MDD patients, we were interested in determining whether a similar relationship exists between baseline HRV (i.e., RSA and LF-HRV) and treatment response in BDD patients. Third, we were interested in determining whether HRV (i.e., RSA and LF-HRV) changes during the course of treatment of our BDD patients, and if so, would responders differ from non-responders with respect to these components of HRV at the end of treatment.

## Materials and Methods

### Study Population

The study was approved by the Institutional Review Board (IRB) of Loyola University Medical Center and was conducted according to the principles of the Declaration of Helsinki. Males and females 18–65 years of age who met DSM-IV criteria for BD I or BD II without any other psychiatric diagnoses, who were otherwise physically healthy and mentally capable to give informed consent, were considered as candidates. We selected BDD subjects whose depression had failed to remit following at least one adequate trial with an antidepressant, or who were experiencing a breakthrough depressive episode in spite of being maintained on a mood stabilizer and/or an atypical antipsychotic agent. As a condition to enrolling in the study, manic/hypomanic symptoms had to have responded adequately to a mood stabilizer and/or antipsychotic. Subjects were maintained on a mood stabilizer and/or atypical antipsychotic throughout the study. Since we used celecoxib as augmenting agent in one arm of this study, subjects who were being maintained on lithium at the time of screening could not be included due to a potential adverse interaction with celecoxib. If they qualified for the study and were agreeable to having their lithium replaced with a different mood stabilizer, they were enrolled. A minimum score of 18 on their 17-item Hamilton Depression Scale (HAMD-17) was required for study admission. Other Axis I diagnoses, active suicidality, uncontrolled hypertension, dyslipidemia, or diabetes mellitus, and history of smoking or substance abuse in the preceding 6 months. History of heart disease or autoimmune disorder was exclusion criteria. Subjects had to be free of any source of active or chronic inflammation. Female subjects could not be pregnant, lactating, or taking oral contraceptives. Screening blood samples were obtained to determine complete blood count, complete metabolic panel, lipid profile, thyroid function, and urinalysis (including pregnancy test). The presence of any clinically significant abnormalities excluded the prospective participant. Sixty-five treatment-resistant BDD patients who met the inclusion/exclusion criteria and successfully completed the baseline evaluations were randomized into one of the two treatment arms: escitalopram + placebo or escitalopram + celecoxib. The primary hypothesis underlying this study was to determine whether modulation of the inflammatory response by co-administration of a specific cyclooxygenase (COX-2) inhibitor would reverse treatment resistance and lead to a better treatment outcome. A total of 37 subjects had complete sets of HRV data to allow meaningful statistical analyses. Their demographic data is shown in Table 1.

**Table 1:** Demographic characteristics of BPD patients versus healthy control (HC) subjects.

	BPD subjects	HC subjects	*p*/χ^2^ value
Study participants	37	36	
Age (±SD)	42.5 (11.8)	39.3 (13.8)	0.28
BMI (±SD)	31.3 (6.4)	26.7 (5.9)	0.003
Female	64.9%	63.9%	0.93
Caucasian	64.9%	75.0%	0.35
Non-Caucasian	35.1%	25.0%	

To quantify the degree to which study patients were treatment resistant we used the Maudsley Staging Method to obtain a resistance score. The scale utilizes a variety of factors to quantify treatment resistance in depression, including duration of depressive symptoms, symptom severity, number of treatment failures, and whether or not the patient had received psychopharmacological augmentation or ECT (26, 27). Each patient was assigned a score with a range of 3 (minimal resistance) to 15 (maximal resistance). Seventy percent of our subjects had scores between 5 and 8, while 30 % had scores between 9 and 13.

### HC Subjects

Eligible HC subjects were recruited by advertising and posting of IRB approved flyers. To determine eligibility, identical procedures were used as for the BDD group including a psychiatric diagnostic structured interview and routine laboratory tests. Main exclusion criteria were any medical, inflammatory, or mental illness and substance use (also among first degree relatives). Pregnant or lactating females were excluded. Their HAMD-17 and Beck Depression Inventory scores had to be less than 5. Thirty-six subjects were enrolled resulting in a BDD/HC ratio of about 1:1. Their demographic data are shown in Table 1.

### Study Design

At the screening visit, subjects underwent a psychiatric interview to establish the diagnosis of treatment-resistant BDD. Subjects who met the screening criteria and signed the IRB-approved consent form underwent comprehensive assessments in order to quantify depression and associated symptoms. Patients then underwent a 2-week washout of their current antidepressant (4 weeks for fluoxetine). After the washout, subjects entered a 1-week run-in phase and received on a single-blind basis escitalopram placebo + celecoxib placebo. The purpose of this run-in was to identify placebo responders. Subjects who continued to meet eligibility criteria at the subsequent baseline visit, were randomized to receive on a double-blind basis escitalopram (beginning at 10 mg/day), +celecoxib (fixed at 400 mg/day), or celecoxib placebo. Escitalopram doses were optimized based on efficacy and tolerability over the first 4 weeks of active treatment but did not exceed a daily dose of 20 mg; no further dose adjustments could be made during the final 4 weeks of the study. Subjects were randomized according to a fixed assignment ratio of 1:1 (escitalopram + celecoxib or escitalopram + placebo). Assignment to groups was based on a pharmacy generated randomization code. The randomization code was kept in the pharmacy and could only be broken if a serious adverse reaction occurred. All study medications were prepared by the pharmacist and were handed to study subjects at each visit. They were instructed to return the empty vials at each visit to determine any amount of unused medication and hence failure to comply.

No discontinuation of medication was permitted throughout the study. Enrolled patients received no other form of therapy for the duration of the study. Follow-up blood draws and assessments using both self-rating and clinician-administered depression and anxiety scales were performed at weeks 0, 1, 2, 4, and 8.

Subjects had to complete at least 6 weeks of active treatment to be regarded as completers. If a subject chose to withdraw from the study on or after 6 weeks of treatment, s/he was expected to complete the end-of-study assessments at that time. Those results were carried forward for the purpose of data analysis.

### Collection of HRV Data

Patients were assessed for HRV at weeks 0 and 8 using the SphygmoCor^®^ CPVH system. This test was always carried out between 8 and 11 h in the morning and always in the same examination room to minimize environmental factors and diurnal fluctuations in ANS function. The patient was asked to recline on the examination table and a three-lead ECG was attached to the chest of the subject who had to rest for 10–15 min before the ECG recording was started. ECG data were collected over a 15-min period to ensure consistency in data collection. There is significant evidence that short-term HRV measurements (30 min or less) are stable over a significant period of time as compared to 24-h measurements *via* a Holter monitor (28, 29).

### Inter-Beat-Interval Editing and Analysis

Data collected for HRV quantification are subject to artifacts that are related to the function of the ECG. The components of HRV (i.e., RSA and LF-HRV) were calculated from a time series generated by the times between sequential heartbeats (i.e., the time in millisecond between sequential R-wave in the ECG) over a period of 10–15 min. This time series consists of several hundred values that correspond to individual inter-beat-intervals (IBIs). Physiological mechanisms, both related and unrelated to RSA or LF-HRV, can contribute to this time series by distorting the accuracy of the R-wave detection. Influences from a spurious decrease in R wave amplitude, a random abnormally large T wave, single PACs/PVCs, or even patient activity must be removed from the time series before RSA and LF-HRV can be reliably quantified.

To deal with potential anomalies through artifact or ventricular arrhythmia (e.g., RSA is an atrial rhythm and represents the time course of the vagal influence on the sinoatrial node), a software package was used to correct for any of the artifacts in the data collected (30). Editing involved integer arithmetic to adjust the time series by adding IBIs when false invalid intervals occurred and dividing IBIs when R wave detections were missed. These decisions were guided by inspection of the ECG. In order to preserve an accurate representation of the neural regulation of the heart, data were only accepted if less than 5% of the data needed to be corrected.

After visual scanning and editing, the data were analyzed using CardioBatch Software (31). CardioBatch is a program that was created as a companion program to CardioEdit to quantify RSA and LF-HRV based on previously developed procedures by Dr. Stephen Porges (32). Fifteen minutes of ECG data were collected for each individual. Values for heart rate and RSA were calculated in sequential 30-s epochs and then averaged across the 15-min period. RSA and LF-HRV values for each epoch were transformed to their natural logarithmic values to conform to the distributional requirements for parametric analyses (12, 33).

### Statistical Analyses

Consistent with the literature that documents HRV decreases with advancing age (34, 35), both the BDD and HC groups exhibited a significant negative relationship between HRV and age. Additionally, the BDD group had a significantly higher BMI than the HC group. To assess the contribution of potential confounding variables (e.g., age, sex, body-mass-index), analyses of covariance (ANCOVA) were used to remove potential confounding effects of age, sex, and ethnicity when comparing RSA and LF-HRV for HC and BD subjects at baseline (Table 1). In addition, heart period (HP) was analyzed. HP represents the average interval between heartbeats measured in milliseconds. HP period increases in duration when heart rate decelerates and decreases in duration when heart rate accelerates. Each patient was assigned a Maudsley score with a range of 3 (minimal resistance) to 15 (maximal resistance). A Pearson’s correlation was then run between severity of treatment resistance and BL RSA, LF-HRV, and HP.

Repeated measures ANCOVA were conducted to evaluate potential differences in the autonomic parameters (i.e., RSA, LF-HRV, HP) at baseline and week 8 in both the escitalopram + celecoxib group and the escitalopram + placebo group, with controls for week 8 escitalopram dosage and treatment response.

Treatment response in study completers was classified as (a) no response to treatment (less than 50% reduction in HAM-D score from baseline); (b) partial response to treatment (greater than 50% reduction but end-of-study HAM-D score greater than 7); (c) remission (greater than 50% reduction and end-of-study HAM-D score of less than 7). It is generally accepted that for a major depressive episode, patients who have had a partial response to antidepressant treatment are at much higher risk of experiencing physical and mental dysfunction in comparison to patients who achieve remission (36). For purposes of the present analyses, no response and partial response were grouped into the non-response category. ANCOVA compared autonomic parameters at baseline between treatment responders and non-responders.

Level of significance for the analyses was set at *p* < 0.05. *p-*Values between 0.05 and 0.10 are reported as trends to be investigated in future studies with larger sample sizes (i.e., greater statistical power).

## Results

**Figure 1:**
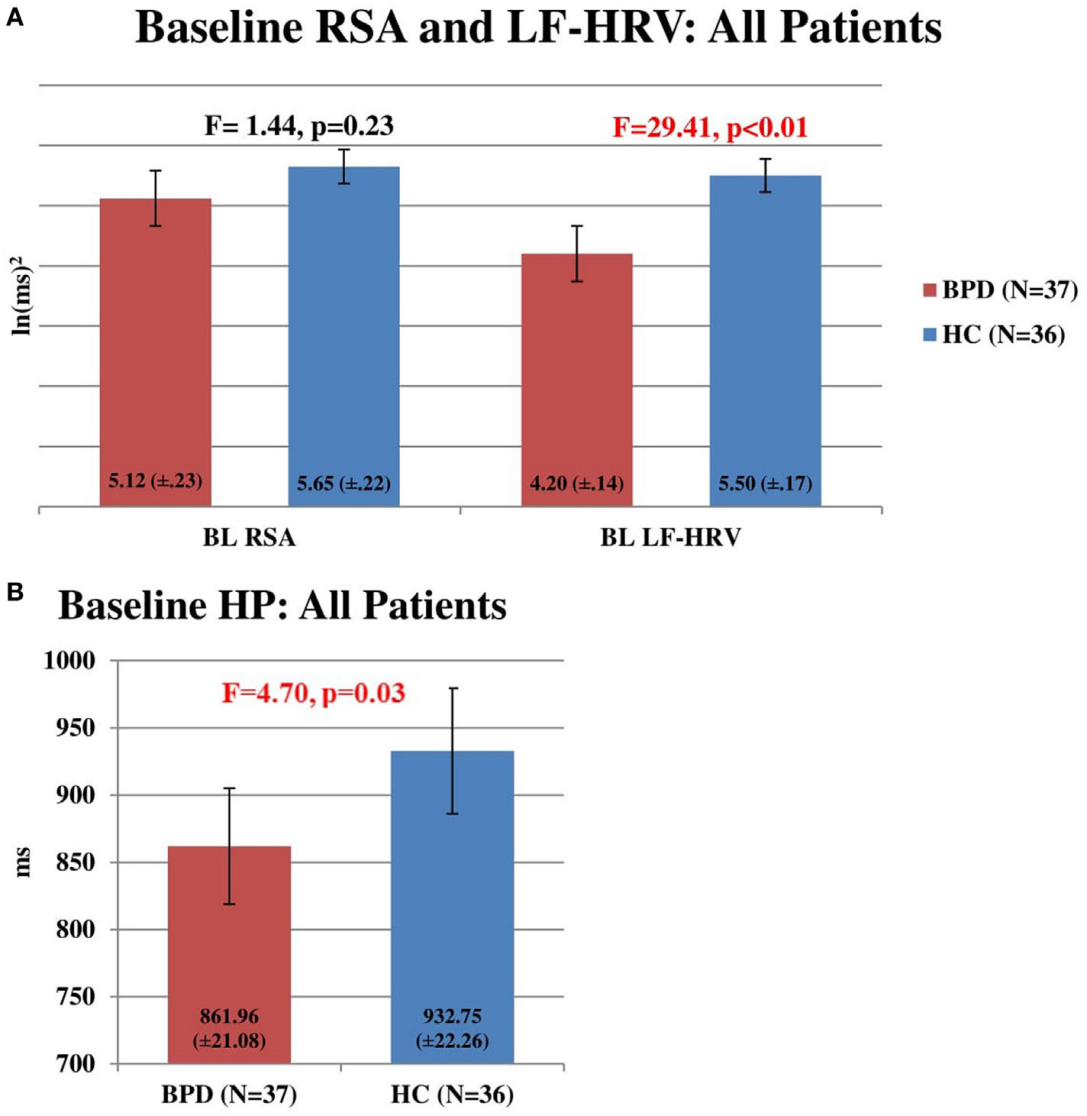
**(A)** Baseline respiratory sinus arrhythmia (RSA) and LF-heart rate variability (HRV) in all patients. Comparison of baseline RSA and LF-HRV in HC subjects (*n* = 36) and bipolar disorder (BD) patients (*n* = 37). No significant difference was found between RSA in HC subjects (5.65, SEM = 0.22) and BPD patients (5.12, SEM = 0.23) (*F* = 1.44, *p* = 0.23). Baseline LF-HRV was significantly higher in HC subjects (5.50, SEM = 0.17) than in BD patients (4.20, SEM = 0.14) (*F* = 29.41, *p* < 0.01). **(B)** Baseline heart period (HP) in all patients. Comparison of baseline HP in HC subjects (*n* = 36) and BD patients (*n* = 37). Baseline HP was significantly higher in HC subjects (932.75 ms, SEM = 22.26) than in BD patients (861.96 ms, SEM = 21.08) (*F* = 4.70, *p* = 0.03).

Baseline RSA did not distinguish BDD patients (*n* = 37) from HC subjects (*n* = 36) (*F* = 1.44, *p* = 0.23). However, baseline LF-HRV of BDD patients (*n* = 37) was significantly lower than HC subjects (*n* = 36) (*F* = 29.41, *p* < 0.01). Also, baseline HP of BDD patients (*n* = 37) was significantly shorter than HC subjects (*n* = 36) (*F* = 4.70, *p* = 0.03) (Figures 1A,B).

BDD patient Maudsley scores were significantly and negatively correlated with baseline RSA (*r* = −0.458, *p* < 0.01). Maudsley scores also tended to be negatively correlated with baseline LF-HRV (*r* = −0.255, *p* = 0.127) and baseline HP (*r* = −0.274, *p* = 0.101), although the relationship did not reach statistical significance (Table 2).

**Table 2:** Correlations between severity of treatment resistance and heart rate variability (HRV) parameters.

	BL RSA	BL LF-HRV	BL HP
Maudsley score	*r* = −0.458, *p* < 0.01	*r* = −0.255, *p* = 0.13	*r* = −0.274, *p* = 0.10

*BL, baseline; RSA, respiratory sinus arrhythmia; LF, low frequency; HRV, heart rate variability; HP, heart period*.

No significant differences in the autonomic parameters were found between BDD patients receiving the escitalopram + celecoxib combination (*n* = 21) and the escitalopram + placebo combination during the baseline assessment (*n* = 14) (data not shown).

**Figure 2:**
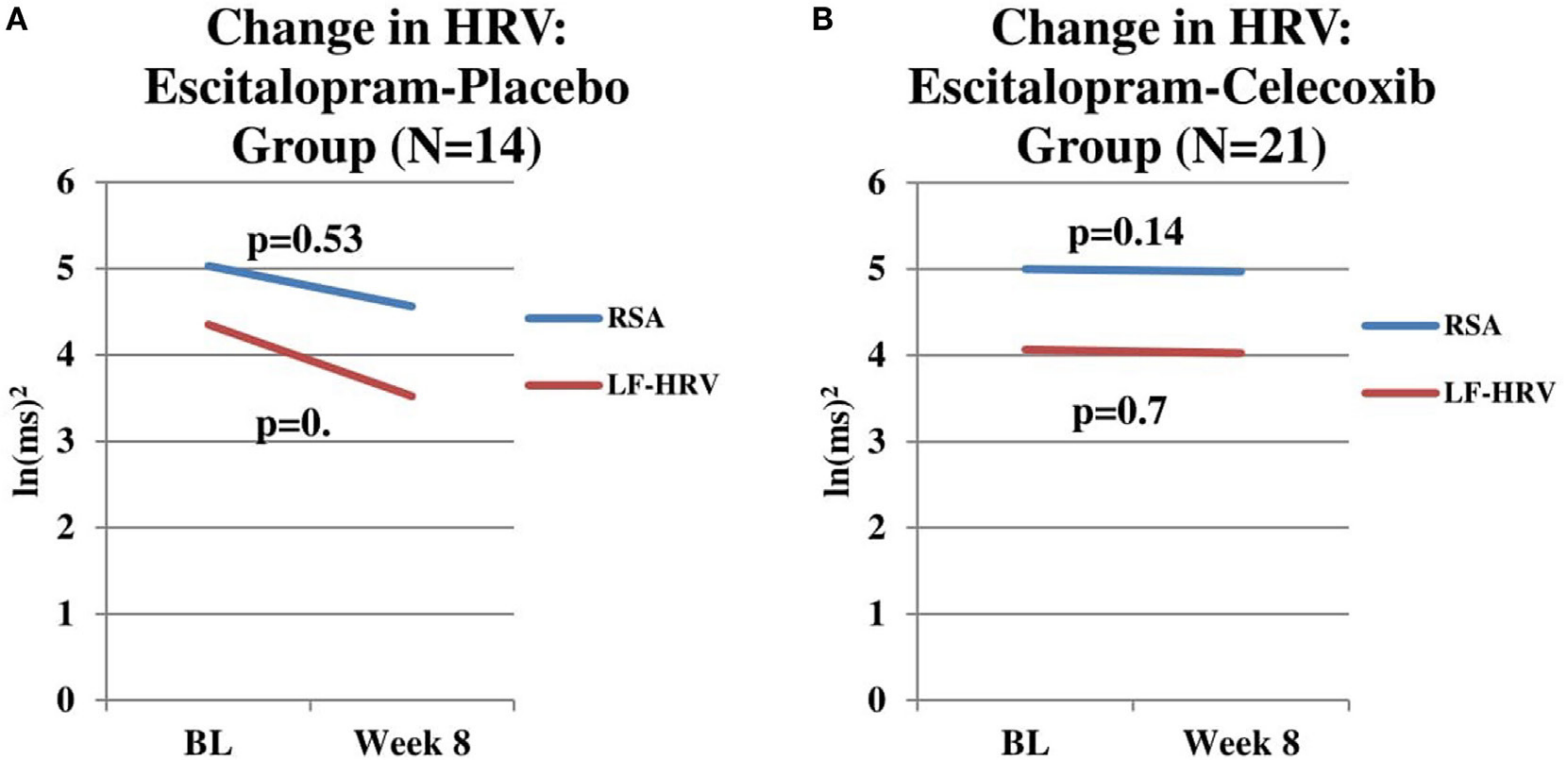
**(A)** Change in respiratory sinus arrhythmia (RSA) and LF-heart rate variability (HRV) in escitalopram-placebo group. Change in RSA and LF-HRV from baseline to end-of-study in patients receiving Escitalopram-placebo combination (*n* = 14). No significant changes in RSA (*p* = 0.54) or LF-HRV (*p* = 0.40) were found. **(B)** Change in RSA and LF-HRV in escitalopram–celecoxib group. Change RSA and LF-HRV from baseline to end-of-study in patients receiving escitalopram–celecoxib combination (*n* = 21). No significant changes in RSA (*p* = 0.14) or LF-HRV (*p* = 0.70) were found.

For the escitalopram + placebo group (*n* = 14), RSA did not change significantly from baseline to week 8 after controlling for change in escitalopram dosage (*F* = 0.42, *p* = 0.53) and after considering treatment response (*F* = 2.89, *p* = 0.12) (Figure 2A). LF-HRV did not change significantly from baseline to week 8 after controlling for change in escitalopram dosage (*F* = 0.54, *p* = 0.48) and after considering treatment response (*F* = 1.38, *p* = 0.27) (Figure 2A). HP did not change significantly from baseline to week 8 after controlling for change in escitalopram dosage (*F* = 0.00, *p* = 0.96) and after considering treatment response (*F* = 0.00, *p* = 0.98) (data not shown).

For the escitalopram + celecoxib group (*n* = 21), baseline RSA for patients who were deemed to be end-of-study treatment responders (*n* = 13) was not significantly different than baseline RSA for patients who were deemed to be end-of-study treatment non-responders (*n* = 8) (*F* = 2.06, *p* = 0.17). No significant differences were found between responders and non-responders for baseline LF-HRV (*F* = 3.16, *p* = 0.10) and baseline HP (*F* = 0.04, *p* = 0.85) (data not shown). Visual inspection of these two figures indicates a flat time course of RSA and LF-HRV in the escitalopram + celecoxib group whereas the time course of change in the escitalopram + placebo group indicates a possible trend toward reduction in both components. While neither time course reached statistical significance, it is intriguing to speculate that the celecoxib combination might exert a “protective effect” against RSA and LF-HRV reduction, the latter being possibly associated with one or more of the concomitant medications, these patients were exposed to prior to and during the current study. Clearly an extended time course of observation and a larger sample size would be needed to investigate such a potential protective effect of the anti-inflammatory agent.

For the escitalopram + celecoxib group (*n* = 21), RSA did not change significantly from baseline to week 8 after controlling for change in escitalopram dosage (*F* = 2.36, *p* = 0.14) and between treatment response groups (*F* = 0.09, *p* = 0.76) (Figure 2B). LF-HRV did not change significantly from baseline to week 8 after controlling for change in escitalopram dosage (*F* = 0.14, *p* = 0.72) and after considering treatment response (*F* = 0.19, *p* = 0.67) (Figure 2B). HP did not change significantly from baseline to week 8 after controlling for change in escitalopram dosage (*F* = 2.18, *p* = 0.16) and after considering treatment response (*F* = 0.16, *p* = 0.70) (data not shown).

## Discussion

As illustrated in Figure 1, at baseline, when our BDD patients were at least moderately depressed, but not manic or hypomanic, relative to HC, they had significantly lower LF-HRV, and HP and a trend toward lower RSA. There were negative correlations between treatment resistance severity and baseline RSA, LF-HRV, and HP, with significance reached in the negative relationship between treatment resistance severity and RSA. This significant negative correlation supports the assumption that treatment-resistant BDD may account, at least in part, for reduced RSA. In this context, the role of possible effects of multiple medication trials and specific medications with anticholinergic properties must also be considered. Decreased HRV has been reported in bipolar patients during the manic phase in some studies (37, 38). However, these findings were not confirmed in a more recent study (24, 25) in which the investigators found increased HRV during manic states compared with depressive and euthymic states using a longitudinal study design with repeated measurements. Additionally, these authors reported an inverse relationship between HRV and the severity of depressive symptoms and a positive association between HRV and the severity of manic symptoms. In another study of bipolar patients studied during a euthymic state, Cohen et al. reported that time domains of HRV (HR, HP) were decreased compared to HCs; however, HF-HRV (also referred to as “vagal tone”) was significantly *increased* (39). A recent study comparing bipolar II depressed patients to unipolar major depressed patients and HCs found that BD patients had a significantly lower vagal tone than both HCs and unipolar major depressed patients (40). Specifically, in bipolar I patients, one study found that subsyndromal BD patients had significantly lower HRV parameters than HCs (41). Faurholt-Jepsen et al. (24, 25) recently published data from a systematic and extensive meta-analysis of 15 studies comprising a total of 2,534 patients and showed that HRV is reduced in BD patients compared to HC subjects. They further commented that the discrepant findings among the published studies could be due, at least in part, to factors unrelated to BD *per se*, notably, the heterogeneity of the disorder, phase of the illness at the time of study, sample sizes, and methods used. To this list of variables that must be controlled in future studies, a detailed list of all medications the patient is receiving at the time of the study should be included with special consideration to agents with established anticholinergic activity as well as known noradrenergic properties.

To our knowledge, ours is the first study to find significant decreases in both time and frequency domain parameters of HRV in bipolar I and II depressed patients in comparison to HC subjects. Our findings are suggestive of the following. First, the directionality of both RSA and LF-HRV is supportive of the Polyvagal Theory (11), which proposes that there are two vagal inputs to the heart. The myelinated portion of the vagus nerve originates in the nucleus ambiguus and is responsible for the effects of RSA, whereas the unmyelinated portion of the vagus originates in the dorsal motor nucleus and contributes to bradycardia and the low-frequency (slow) bandwidth of HRV (11). If LF-HRV reflected any sympathetic activity, we would expect to see either no change or possibly even an increase in BDD patients, but the opposite was actually detected in our study. This finding is consistent with similar findings in MDD patients and frequency domain HRV measurements (42). It reinforces the need for further exploration of the physiological mechanism behind LF-HRV and is consistent with the evidence that there is no neural basis to interpret LF-HRV as an index of sympathovagal balance (43).

Current evidence linking depression and HRV is inconclusive and is largely based on the majority of research done in MDD subjects. Initially, MDD appeared to be associated with decreased HRV, and no effect of Selective serotonin reuptake inhibitors (SSRI’s) on HRV was detected over a 3–6 week trial period (44). This finding is similar to the lack of effect found in our earlier study with MDD patients (see text footnote 1). However, data obtained over a 2-year period of observation show a significant decrease in HRV in MDD patients receiving antidepressants in comparison not only to HC subjects but also to MDD patients not on antidepressants (45). More recent research has focused on specific medications, and it appears that tricyclic antidepressants (TCAs) are most robust in reducing HRV, followed by serotonin/norepinephrine reuptake inhibitors (46, 47). SSRIs have the least effect on cardiac function and may even *decrease* cardiac sympathetic impact (46). In the present study with BDD patients, we did not observe a change in HRV parameters after 2 months of exposure to escitalopram, and this observation is consistent with the current literature. This finding was obtained with or without the addition of celecoxib to the treatment regimen.

While there is limited evidence linking BDD to HRV, the link between specific medications and vagal tone may offer a glimpse into the findings of our study. TCAs have classically been associated with anticholinergic side effects (e.g., dry mouth, constipation, blurry vision, urinary retention), as well as cardiotoxicity and neurotoxicity in cases of overdose. It is not surprising then that TCAs could exert a deleterious effect on the heart (48). Other medications in psychiatric populations that have been routinely associated with anticholinergic side effects are antipsychotics, particularly, the typical antipsychotics, but also atypical antipsychotics to a degree. Of our 37 patients for whom we have complete HRV data, 10 were placed on a bipolar medication regime throughout the study that included an atypical antipsychotic, while seven were maintained only on an atypical antipsychotic for mood stabilization. The remaining patients were maintained only on a mood stabilizer, most commonly lamotrigine or valproic acid. Atypical antipsychotics most commonly used were quetiapine and aripiprazole, and, less frequently, ziprasidone, olanzapine, and risperidone. Many of our patients had been prescribed several of these medications throughout the course of their lives. We chose not to compare baseline HRV parameters between patients on different mood stabilization medication due to the confounding variables of dosage variations, duration of treatment, and lack of adequate sample size.

Growing evidence indicates that antipsychotic medications can exert an effect on HRV in psychiatric patients. A recent meta-analysis established that clozapine is associated with a significant reduction in HRV (49). Another study determined that, in schizophrenic patients taking olanzapine, patients who gained a significant amount of weight after 1 month had a significantly lower HRV than patients who did not gain weight (50). These findings are not surprising, considering these two atypical antipsychotic agents are most commonly associated with metabolic side effects and are, therefore, more likely to have deleterious effects on the heart (51). These negative effects on HRV also appear to be dose-dependent, with higher doses being associated with further decreases in HRV (52). There have been other findings that atypical antipsychotics decrease HRV, but these papers do not specify which specific medications were used (53, 54). It is worth speculating, however, that atypical antipsychotics with a lower anticholinergic profile may be less prone to reduce HRV parameters. Clozapine, olanzapine, and, to a less degree, quetiapine, have all been shown to have anticholinergic side effects (55). These anticholinergic effects can be hypothesized to decrease vagal tone, reduce overall HRV, and contribute to increased cardiac morbidity and mortality that is often noted in patients taking antipsychotic medication.

Anticholinergic properties of psychiatric medications have resulted in unwanted, unpleasant, or even dangerous side effects for psychiatric patients. There lies the possibility that these properties may also hinder a full treatment response as well. In our pervious study, MDD patients who did not respond to a 3-month trial of either escitalopram or quetiapine monotherapy had significantly lower vagal tone than patients who did respond (see text footnote 1). This finding was not replicated in our BDD patient study, but the theoretical implications are still worth noting. Many of the second line agents used for depression in both MDD and BDD are strongly anticholinergic, including TCAs and atypical antipsychotics, such as quetiapine and olanzapine. Thus, what may actually be happening is that we are making a certain subset of these otherwise treatment-resistant patients *worse*, by giving them medications that exacerbate possible inherent mechanisms responsible for their underlying treatment resistance.

What are the clinical implications of HRV in BDD patients? The primary aim of the BDD study was to assess the role of inflammation in the pathophysiology of bipolar depression and determine if anti-inflammatory adjunctive treatment would aid in remission of depressive symptoms in BDD patients. Not only did patients receiving the escitalopram + celecoxib combination experience a significant decrease in depressive symptoms over a 2-month period in comparison to the escitalopram + placebo group, but they achieved remission much faster than the placebo group ((56); Halaris et al., in preparation).

One possible mechanism underlying the pathophysiology of treatment resistance in either MDD or BDD can be the associated pro-inflammatory state and ANS dysregulation with associated diminution in vagal tone. A decrease in vagal tone likely leads to dysregulation of the body’s inflammatory response mediated, in part, by the cholinergic anti-inflammatory pathway (57). It has been demonstrated that efferent vagal fibers originating in the dorsal motor nucleus can modify the release of inflammatory cytokines, such as TNFα, from macrophages, thereby preventing over-activation of the inflammatory process without inducing immunosuppression (58). Loss of vagal tone, therefore, may be crucial to understanding the pro-inflammatory status associated with MDD and BD that has been described in the literature (59, 60). If parasympathetic tone can be maintained at HC levels, reflecting a physiological degree of inflammatory response, antidepressant drug action may proceed unhindered ultimately leading to remission. In our study, we did not see a change in HRV over the course of the study in either the combination treatment group or the placebo group. Celecoxib co-administration modulated inflammation as reflected in significant reduction is specific pro-inflammatory mediators, thereby facilitating and even enhancing the antidepressant efficacy of escitalopram. However, this adjunctive anti-inflammatory treatment did not produce any beneficial cardiovascular effects within the time frame of our study ostensibly due to the anticholinergic properties of the concomitant psychotropic medications used in the present study. Nevertheless, these findings suggest an additional and intriguing theory about the mechanism(s) of treatment resistance and its relationship to both depression and CVD.

## Future Studies

Although much time and research have been devoted to distinguishing the depressive phase of BD from MDD, a reliable diagnostic distinction often poses serious challenges to the clinician (61). One potential avenue is to utilize HRV assessment and domain analysis as a diagnostic and prognostic biomarker. Recent evidence suggests that acute HRV measurements can be obtained by only 120 s of ECG recordings (62). Indeed, a recent study compared patients with bipolar II depression and patients with unipolar depression and found significantly lower HF-HRV and a higher LF/HF ratio (used as a measurement of sympathetic tone) in bipolar II depressed patients as compared to unipolar depressed patients (40). Therefore, future studies should take into consideration the length of prior exposure, if any, to pharmacological agents, both psychiatric and non-psychiatric. It will also be interesting to determine if anti-inflammatory medication utilizing selective COX-2 inhibitors can exert beneficial effects on HRV over a longer treatment period. Once the possible effects of pharmacologic agents on HRV domains have been fully clarified, wide utility of HRV as a biomarker will be justified.

## Limitations of the Study

One limitation to our study is the small sample size. Several of our trending variables might have reached statistical significance with a larger patient and/or HC population. Specifically, given the small heterogeneous sub-groups (age, ethnicity, BMI), it may not be viable within this sample to investigate whether components of HRV at baseline (e.g., RSA and LF-HRV) are related to current episode length and/or previous episodes. Although this does not detract from the importance of our findings, reproducibility with a larger population would be necessary to confirm those findings that did not reach statistical significance. As in any study relying on the collection of data over time, several limitations are to be noted. Current mood state of the patient could have influenced the assigned scores in both the self-assessment and rater-administered scales. To minimize such effects, consistent provider–patient pairings were kept over the course of the study to minimize interobserver bias and to allow the patient to receive a consistent level of care. The fact that our patients had to be fully stabilized on mood stabilizers before being administered antidepressant medication may have blunted any changes in HRV during the course of treatment. In addition, the relatively short duration of our recording (15 min) in the resting state precluded the opportunity to assess HRV during both rest and activity and thereby provide a better measure of cardiac resilience.

## Ethics Statement

This study was carried out in accordance with the recommendations of the Declaration of Helsinki and Institutional Review Board with written informed consent from all subjects. All subjects gave written informed consent in accordance with the Declaration of Helsinki. The protocol was approved by the Institutional Review Board of Loyola University Stritch School of Medicine/Loyola University Medical Center.

## Author Contributions

AH designed the study, wrote the protocol, and oversaw the preparation of the manuscript. BH actively participated in data collection, data analyses, literature search, and preparation of all drafts of the manuscript. BB contributed to data management and data analyses. DD participated in the clinical portions of the study and data collection. KH performed data analyses. SP oversaw data analyses, contributed to manuscript preparation, and acted as a consultant to the co-investigators.

## Conflict of Interest Statement

The authors declare that the research was conducted in the absence of any commercial or financial relationships that could be construed as a potential conflict of interest.

## Footnotes

^1^Hage B, Sinacore J, Heilman K, Porges S, Halaris A. Heart rate variability predicts treatment outcome in major depression. *Psychiatry Clin Neurosci* (under review).
